# An open, multi-centre, phase II clinical trial to evaluate the efficacy and safety of paclitaxel, UFT, and leucovorin in patients with advanced gastric cancer

**DOI:** 10.1038/sj.bjc.6603225

**Published:** 2006-06-27

**Authors:** Y Chao, C P Li, T Y Chao, W C Su, R K Hsieh, M F Wu, K H Yeh, W Y Kao, L T Chen, A L Cheng

**Affiliations:** 1 Cancer Center, Taipei Veterans General Hospital, Taipei, Taiwan; 2 Central Clinic Hospital, Taipei, Taiwan; 3 National Yang-Ming University School of Medicine, Taipei, Taiwan; 4 Division of Gastroenterology, Department of Medicine, Taipei Veterans General Hospital, Taipei, Taiwan; 5 Division of Hematology and Oncology, Tri-Service General Hospital, National Defense Medical Center, Taipei, Taiwan; 6 Department of Internal Medicine, National Cheng Kung University Hospital, Tainan, Taiwan; 7 Division of Hematology and Oncology, Department of Internal Medicine, Mackay Memorial Hospital, Taipei, Taiwan; 8 Department of Internal Medicine, Chung Shan Medical University Hospital, Taichung, Taiwan; 9 Department of Oncology, National Taiwan University Hospital, Taipei, Taiwan; 10 Cancer Research Center, National Taiwan University College of Medicine, Taipei, Taiwan; 11 Division of Oncology and Hematology, Department of Internal Medicine, Far Eastern Memorial Hospital, Taipei, Taiwan; 12 Institute of Cancer Research, National Health Research Institutes, Taipei, Taiwan; 13 Department of Internal Medicine, Kaohsiung Medical University Hospital, Kaohsiung, Taiwan

**Keywords:** paclitaxel, UFT, leucovorin, gastric cancer

## Abstract

The aim of the study was to evaluate the response rate and safety of weekly paclitaxel (Taxol®) combination chemotherapy with UFT (tegafur, an oral 5-fluorouracil prodrug, and uracil at a 1 : 4 molar ratio) and leucovorin (LV) in patients with advanced gastric cancer. Patients with histologically confirmed, locally advanced or recurrent/metastatic gastric cancer were studied. Paclitaxel 1-h infusion at a dose of 100 mg m^−2^ on days 1 and 8 and oral UFT 300 mg m^−2^ day^−1^ plus LV 90 mg day^−1^ were given starting from day 1 for 14 days, followed by a 7-day period without treatment. Treatment was repeated every 21 days. From February 2003 to October 2004, 55 patients were enrolled. The median age was 62 years (range: 32–82). Among the 48 patients evaluated for tumour response, two achieved a complete response and 22 a partial response, with an overall response rate of 50% (95% confidence interval: 35–65%). All 55 patients were evaluated for survival and toxicities. Median time to progression and overall survival were 4.4 and 9.8 months, respectively. Major grade 3–4 toxicities were neutropenia in 25 patients (45%) and diarrhoea in eight patients (15%). Although treatment was discontinued owing to treatment-related toxicities in nine patients (16%), there was no treatment-related mortality. Weekly paclitaxel plus oral UFT/LV is effective, convenient, and well tolerated in treating patients with advanced gastric cancer.

Gastric cancer is the second leading cause of cancer death worldwide (Roder, 2002). The prognosis is generally poor, with an overall 5-year survival of approximately 20% in most countries. The median survival time of patients who presented with advanced or metastatic diseases and received best supportive care was only 3–5 months (Verdecchia *et al*, 2003). For decades, gastric cancer has been considered as a chemo-resistant tumour. With the introduction of novel drug administration schedules and the emergence of new chemotherapeutic agents, modern systemic chemotherapy could achieve an objective response rate of 30–60% in advanced gastric cancers (Schoffski, 2002). The overall survival (OS) also improved to a range between 9 and 11 months; however, significant treatment-related toxicities were usually inevitable (Ohtsu, 2005). New treatments with better therapeutic index are needed to improve the outcome.

Weekly infusion of high-dose 5-fluorouracil (5-FU)/leucovorin (LV), the HDFL regimen, is an effective chemotherapy regimen for advanced gastric cancer, with a response rate of 33–48% and a median survival of 9–10 months, and the haematological toxicities are minimal (Hsu *et al*, 1997; Lin *et al*, 1999). It can be safely applied even to patients with a poor general condition (Yeh and Cheng, 1998; Lee *et al*, 2002). For its efficacy and low-toxicity profile, weekly HDFL *per se* and HDFL-based new combination regimens have been extensively evaluated for the treatment of patients with advanced gastric cancer in Taiwan (Lin *et al*, 2001; Chen *et al*, 2002; Chao *et al*, 2004; Yeh *et al*, 2005). However, the implantation of a central venous catheter is inevitable for these treatments.

UFT, a combination of tegafur (a prodrug of 5-FU) and uracil in a 1 : 4 molar ratio, is a second generation of oral fluoropyrimidine. Uracil serves as a competitive antagonist for dihydropyrimidine dehydrogenase, the major catabolic enzyme of 5-FU, to enhance the concentration and half-life of 5-FU in the circulation. Pharmacokinetically, orally administered UFT simulates the continuously intravenous infusion of 5-FU (Meropol *et al*, 1996; Ho *et al*, 1998). On a 28-day schedule, oral UFT monotherapy could achieve a 20% response rate in advanced gastric cancer without significant myelosuppression, diarrhoea, and stomatitis (Anderson and Lokich, 1992; Takiuchi and Ajani, 1998).

Paclitaxel, an antimitotic agent that stabilises microtubules, exhibits *in vitro* activity against gastric cancer cells (Caplow *et al*, 1994). Paclitaxel monotherapy, at a dose range of 200–250 mg m^−2^ given as either a 24- or a 3-h infusion every 3 weeks, could achieve a tumour response rate of 17–21% and a median response duration of 6.5 months in advanced gastric cancer (Ajani *et al*, 1998; Ohtsu *et al*, 1998; Yamada *et al*, 2001; Schoffski, 2002; Ohtsu, 2005). The non-overlapping toxicity profile of paclitaxel and infusional 5-FU, and the observation of a schedule-dependent synergism between paclitaxel and 5-FU in human gastric cancer cells (Yeh *et al*, 1998), rationalise such a combination to be evaluated in treating advanced gastric cancers.

We conducted a phase II study of combination chemotherapy of paclitaxel, UFT, and LV to determine the response rate and toxicity profile of this combination in patients with advanced gastric cancer.

## PATIENTS AND METHODS

### Patients

Eligibility criteria of the patients included (1) pathologically confirmed, locally advanced (unresectable), recurrent or metastatic gastric cancer, (2) measurable disease by imaging studies, (3) no prior chemotherapy except postoperative adjuvant chemotherapy that had been administrated more than 12 months before entering into the study, (4) an ECOG (Eastern Cooperative Oncology Group) performance status ⩽2, (5) age greater than 18 years old, and (6) adequate hepatic, renal, and bone marrow functions.

Exclusion criteria included (1) pre-existing peripheral neuropathy, (2) pregnancy, breastfeeding, or women of childbearing potential without adequate contraception, (3) a concurrent or prior malignancy, (4) central nervous system metastases, (5) active infection, and (6) concurrent treatments that might interfere with study evaluation. This study was approved by the ethics committee of all participating institutes and signed informed consent was obtained from all patients.

### Study design

This was a prospective, multi-centre, phase II clinical trial. The primary objective was to evaluate the response rate of weekly paclitaxel combination chemotherapy with UFT and LV in patients with advanced gastric cancer. The secondary objective was to determine the time to progression (TTP), OS, and safety.

### Chemotherapy protocol

Paclitaxel (Taxol®, Bristol-Myers Squibb, Princeton, NJ, USA) was administered as a 1-h continuous intravenous infusion at a dose of 100 mg m^−2^ on days 1 and 8. To reduce the risk of hypersensitivity reactions to paclitaxel, all patients were premedicated with 10 mg of dexamethasone, 300 mg of cimetidine, and 50 mg of diphenhydramine intravenous infusion 30 min before chemotherapy. UFT® (Taiho Pharmaceutical Co. Ltd., Tokyo, Japan) 300 mg m^−2^ day^−1^ and LV (Leucovorin®, Wyeth Farma, SA Madrid, Spain) 90 mg day^−1^ were administered orally starting from day 1 and continuing for 14 days, followed by a 7-day period off treatment. The total daily dose of UFT was determined and rounded to the nearest 100 mg and divided into three doses given 8 h apart. If the total number of tablets could not be evenly divided, the highest dose was given in the morning and lower doses in the afternoon or evening. Cycles were repeated every 3 weeks or upon recovery from toxicities to baseline or grade 1 (except alopecia and anaemia).

### Dose modification

Chemotherapy doses may be reduced or treatment may be delayed for no more than 2 weeks to allow for patients' recovery. The dose of each study drug was reduced stepwise. Level 1 was paclitaxel 80 mg m^−2^, UFT 250 mg m^−2^, and LV 90 mg day^−1^, and level 2 was paclitaxel 60 mg m^−2^, UFT 200 mg m^−2^, and LV 90 mg day^−1^. A maximum of two dose-level reductions were allowed per patient. Any patient who required a reduction of dose to lower than level 2 must be excluded from the treatment protocol. Dose modification was based on haematological toxicity and on non-haematological toxicity. At haematological nadir, if the neutrophil count was <500 mm^−3^ or platelets <25 000 mm^−3^, the next dose was reduced by one level. If a haematology test within 72 h before the next cycle indicated that the neutrophil count was <1000 mm^−3^ or the platelet count was <100 000 mm^−3^, the next cycle should be delayed until haematological recovery. Haematology test should be performed every week to identify the haematological recovery. If recovery was not achieved within 2 weeks from the scheduled date of next cycle, the patient must be taken off study protocol. If haematological recovery was not achieved before day 8 from the scheduled date, then paclitaxel administration was skipped on day 8.

Non-haematological toxicities were graded. Paclitaxel administration was resumed at the same dose level during a cycle upon recovery from toxicities to the baseline or grade 1 (except alopecia and anaemia). If recovery from non-haematological toxicity was not achieved before day 8 from the scheduled date, the paclitaxel dose was skipped on day 8 and the next dose was reduced by one level. For a toxicity greater than grade 2, UFT and LV administration was interrupted. When the toxicity subsided to baseline or less than grade 1, administration was resumed. Treatment was not continued after treatment cycle day 14, regardless of the number of days the drug has been interrupted.

### Evaluation of efficacy and toxicities

Evaluations before chemotherapy included medical history taking, physical examination, complete blood count, blood chemistry, chest X-ray, and computed tomography (CT) scan and/or magnetic resonance imaging (MRI) of abdomen. After starting protocol treatment, a complete blood count was conducted weekly and blood chemistry every 3 weeks. Detailed history, physical examinations, and treatment-related toxicities were recorded weekly. Tumour size was measured by imaging studies (CT and/or MRI) every 6 weeks. Tumour response was evaluated according to the World Health Organization criteria (Miller *et al*, 1981). All subjects with tumour responses (complete response (CR) and partial response (PR)) underwent a confirmatory scan 4 weeks following the initial documentation. Toxicities were graded according to the NCI-Common Toxicity Criteria (version 2) (Trotti *et al*, 2000).

### Statistical analyses

The Simon optimal two-stage design was used (Simon, 1988). The response rate of interest were *P*_0_=30% and *P*_1_=50%. If objective tumour responses were observed in more than five of 15 evaluable patients in the first stage, an additional 31 evaluable patients would be enrolled in the second stage. If there were more than 19 responders at the end of the second stage, this treatment would be considered as effective and deserving further investigation, with an *α*-value of 0.05 and a *β*-value of 0.10. Time to progression was defined as the duration from the date of starting protocol treatment to the date of documented disease progression or death by any cause. Overall survival was defined as the duration from the date of starting protocol treatment to the date of death. Survival was estimated by Kaplan–Meier analyses.

## RESULTS

### Patients and treatment

Between February 2003 and October 2004, 55 patients were enrolled into the study from six medical centres. The major clinicopathologic characteristics of patients are listed in Table 1. The median age of the patients was 62 years (range: 32–82). A total of 343 (median: 6; range: 1–21) cycles of chemotherapy were given. Median relative dose intensity was 98% (range: 70–100%) for paclitaxel, 88% (range: 7–100%) for UFT, and 88% (range: 7–100%) for LV. In total, 96% of the patients received more than 80% of the intended doses of paclitaxel, and 56% received more than 80% of the intended doses of UFT and LV.

### Efficacy

Seven patients were not evaluable for tumour responses: four patients failed to return to the clinic for tumour measurements, two patients were found to have no measurable tumours later, and one patient refused chemotherapy. Among the 48 evaluable patients, the best tumour response was CR in two patients, PR in 22 patients, stable disease in 20 patients, and progressive disease in four patients. The overall response rate was 50% (24 out of 48 patients, 95% confidence interval (CI): 35–65%). The response rates for patients with ⩾80 and <80% of scheduled UFT dose intensity were 57% (17 out of 30 patients) and 39% (8 out of 18 patients), respectively (*P*=0.3715). The median time to tumour response was 3 (range: 2.1–4.4) months. The median duration of tumour response was 5.9 (range: 0.4–12.6) months.

**Figure 1:**
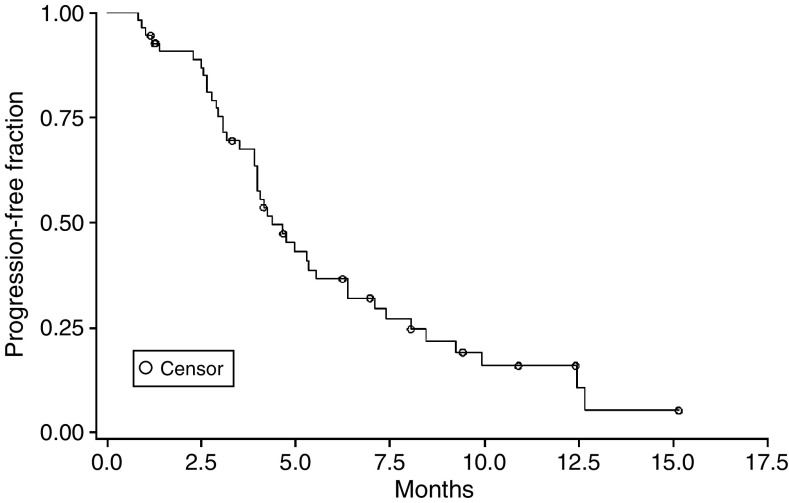
Time to progression of the 55 patients.

**Figure 2:**
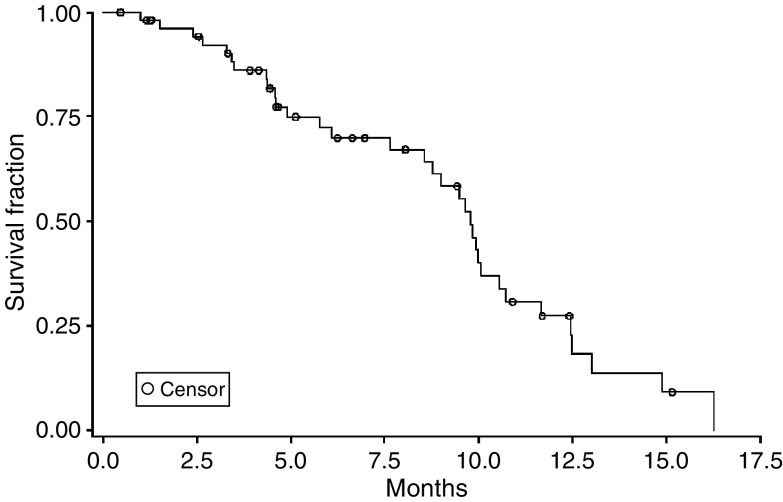
Overall survival of the 55 patients.

On an intention-to-treat analysis, the median follow-up time for the 55 enrolled patients was 11.7 (range: 1.0–30.0) months. The median TTP and OS were 4.4 (95% CI: 4.0–6.5) months and 9.8 (95% CI: 8.6–10.7) months, respectively. The Kaplan–Meier estimated TTP and OS curves are shown in Figures 1 and 2.

### Toxicity

All 55 patients were evaluated for toxicities (Table 2). The most common toxicity was neutropenia, with grade 3–4 neutropenia observed in 45% of the 55 patients evaluable for toxicity. One patient developed neutropenic fever and recovered with appropriate therapy. Grade 3 sensory neuropathy developed in four (7%) patients after a median of five cycles of treatment that invariably improved with discontinuation of chemotherapy. Grade 3–4 diarrhoea was observed in 15% of the patients. Alopecia developed in 40 patients (73%). Grade 3 sinus tachycardia was noted in 5% of the patients. Among them, two occurred during infusion of the paclitaxel and the other was not related to paclitaxel. All recovered spontaneously. A total of 32 (58%) patients had a dose delay during treatment. Dose modification was required in 22 (40%) patients. Treatment-related toxicity resulted in treatment discontinuation in nine (16%) patients, which were associated with paclitaxel-related neuropathy in four patients, a delayed recovery from neutropenia in four patients, and suspicious myocardial ischaemia (chest pain) in one patient. There was no treatment-related mortality.

## DISCUSSION

In this study, we showed that weekly 100 mg m^−2^ of paclitaxel plus daily oral UFT/LV for 2 weeks every 3 weeks is an active combination chemotherapy regimen for patients with advanced gastric cancer. The 50% overall response rate of the evaluable patients (44% on intention-to-treat analyses), a median survival of 9.8 months, and 45% of grade 3–4 neutropenia were compatible with those achieved with topoisomerase I inhibitor-, taxane-, or third generation of oral fluoropyrimidine (i.e. TS-1 and capecitabine)-based doublet chemotherapy in phase II trials (Schoffski, 2002; Ohtsu, 2005), in which the objective tumour response rate and median survival ranged from 40 to 76% and from 9 to 12.5 months, respectively. The median survival of this study was also comparable with those achieved in previous HDFL-based studies in our institutes (Hsu *et al*, 1997; Chen *et al*, 2002; Chao *et al*, 2004), and not inferior to the 6.1–12.0 months of median survival of phase II or III studies of current ‘reference’ regimens for advanced gastric cancers, that is, ECF, FAMTX, FAMTX, ELF, FUP, FOLFOX6, and DCF (Webb *et al*, 1997; De Vivo *et al*, 2000; Vanhoefer *et al*, 2000; Louvet *et al*, 2002; Ajani *et al*, 2005; Ohtsu, 2005).

Recently, the superior therapeutic index of weekly paclitaxel compared to the triweekly schedule has been demonstrated in gastric cancer patients (Ohtsu *et al*, 1998; Yamada *et al*, 2001; Kakeji *et al*, 2005). With a comparable tumour response rate (22–23%) for advanced gastric cancer, the grade 3–4 neutropenia of weekly paclitaxel (80 mg m^−2^, on days 1, 8, and 15 every 4 weeks) and of triweekly paclitaxel (200–210 mg m^−2^, on day 1 every 3 weeks) were 20 and 37–67%, respectively. Weekly paclitaxel plus either infusional 5-FU or oral fluoropyrimidine have become popular investigational combinations for advanced gastric cancers (Ninomiya *et al*, 2005; Takeyoshi *et al*, 2005; Ueda *et al*, 2005; Yeh *et al*, 2005). Among these phase II studies, including the current one, the tumour response rate was quite consistent, ranging from 35 to 50% in chemo-naïve gastric cancer patients (Ninomiya *et al*, 2005; Takeyoshi *et al*, 2005; Yeh *et al*, 2005). The efficacy parameters (objective response, TTP, and OS) and toxicity profiles (esp. grade 3–4 neutropenia) of current regimen were also nearly identical to those observed in the previous paclitaxel plus infusional HDFL trial performed by Yeh *et al* (2005). The advantage of this oral approach is the alleviation of the requirement for and the cost of central venous catheter implantation and the associated inconvenience of infusion pump, which in turn might improve the quality of life of treated patients. Our results suggest that, while in combination with weekly paclitaxel, daily administration of oral UFT/LV might achieve a similar therapeutic index and ultimately replace intermittent infusional 5-FU/LV treatment for advanced gastric cancer.

Recently, Takeyoshi *et al* (2005) showed that paclitaxel 80 mg m^−2^ intravenously on days 1, 8, and 15 every 4 weeks and doxifluridine (5′-dexoy-5-fluorouridine, an intermediate metabolite of capecitabine) 533 mg m^−2^ orally on days 1–5 per week could achieve a tumour response rate of 46% with only 12% of grade 3–4 leucopenia in chemo-naïve patients. Although the results (including the unexpected low incidence of myelosuppression) have to be verified by a large-scale clinical trial, the 5-days-on/2-days-off oral fluoropyrimidine schedule is of interest. A recent pharmacological study on surgical specimens from colorectal cancer patients who had received preoperative 5-days (weekday)-on/2-days (weekend)-off UFT treatment showed maintenance of a relatively high level of intratumoral concentration of 5-FU at 48 h after the last dose of UFT (Sadahiro *et al*, 2001). The schedule was considered to reduce the incidence of adverse events and to improve treatment compliance. Can the modification of UFT administration schedule further improve the therapeutic index of currently weekly paclitaxel plus UFT/LV regimen deserves further exploration.

Grade 3 sinus tachycardia was noted in 5% of the patients. Asymptomatic cardiac disturbances have been reported during paclitaxel infusion (Kamineni *et al*, 2003). Hypersensitivity to paclitaxel or coexistence of underlying diseases may be the aetiologies. The exact cause was unknown. Caution should be exercised in patients with underlying cardiac diseases and more prospective studies are needed to assess its cardiotoxicities.

Nowadays, several novel targeted therapeutic agents such as inhibitors of epidermal growth factor receptor or of vascular endothelial growth factor, when used in combination with chemotherapy, have shown promising activity against gastrointestinal cancers (Chong and Cunningham, 2005). The low-toxicity profiles of such agents also provide an excellent chance to improve the therapeutic index of this active and convenient regimen for advanced gastric cancer patients.

In conclusion, the combination of weekly paclitaxel and oral UFT/LV is an active, outpatient-based chemotherapy regimen with acceptable toxicities. However, for the palliative nature of systemic chemotherapy in recurrent or metastatic gastric cancer, strategies to improve the therapeutic index of the current regimen, that is, modification of drug administration schedule and/or in combination with novel biological targeted agents, should also be further explored.

## Figures and Tables

**Table 1:** Clinicopathologic features of the patients

	**Patient number (%)**
Total patients	55
Age (years), median (range)	62 (32–82)
Sex: male/female	33/22
	
*ECOG performance*	
0	4 (7)
1	45 (82)
2	6 (11)
	
*Treatments for primary tumour*	
No prior therapy	28 (51)
Surgery only	24 (44)
Surgery+adjuvant chemotherapy	2 (4)
Radiotherapy	1 (2)
	
*Disease status*	
Locally advanced	3 (5)
Recurrence/metastasis	52 (95)
	
*Disease sites*	
Liver	29 (53)
Lymph nodes	24 (44)
Peritoneum	12 (22)
Gastrointestinal tract	14 (25)
Bone	1 (2)
Lung	3 (5)
Others	11(20)

ECOG=Eastern Cooperative Oncology Group.

**Table 2:** Percentage toxicity of the paclitaxel, UFT, and LV regimen

	**Patients (*n*=55)**		**Cycles (*n*=343)**	
	**Grade**	**Grade**	**Grade**	**Grade**
**Toxicity**	**1–2**	**3–4**	**1–2**	**3–4**
*Haematological*				
Neutropenia	25^a^	45	36^b^	22
Leucopenia	55	18	52	6
Thrombocytopenia	2	2	1	0.6
Febrile neutropenia	4	2	0.6	0.3
Anaemia	42	9	28	2
				
*Gastrointestinal*				
Nausea	27	0	14	0
Vomiting	20	0	6	0
Diarrhoea	33	15	18	4
Stomatitis	5	0	1	0
Anorexia	35	2	20	0.3
Weight loss	5	0	4	0
Hypoaesthesia	45	7	46	0
				
*Others*				
Cardiac	7	5	1	1
Fever	2	0	0.3	0
Alopecia	73	0	74	0

LV=leucovorin.

a All numbers are percentage of the 55 patients.

b All numbers are percentage of the 343 cycles given.
